# Toward cross‐platform electronic health record‐driven phenotyping using Clinical Quality Language

**DOI:** 10.1002/lrh2.10233

**Published:** 2020-06-25

**Authors:** Pascal S. Brandt, Richard C. Kiefer, Jennifer A. Pacheco, Prakash Adekkanattu, Evan T. Sholle, Faraz S. Ahmad, Jie Xu, Zhenxing Xu, Jessica S. Ancker, Fei Wang, Yuan Luo, Guoqian Jiang, Jyotishman Pathak, Luke V. Rasmussen

**Affiliations:** ^1^ Biomedical Informatics and Medical Education University of Washington Seattle Washington USA; ^2^ Department of Health Sciences Research Mayo Clinic Rochester Minnesota USA; ^3^ Feinberg School of Medicine Northwestern University Chicago Illinois USA; ^4^ Information Technologies and Services Weill Cornell Medicine New York New York USA; ^5^ Department of Population Health Sciences Weill Cornell Medicine New York New York USA

**Keywords:** Clinical Quality Language, common data models, electronic health records, phenotyping

## Abstract

**Introduction:**

Electronic health record (EHR)‐driven phenotyping is a critical first step in generating biomedical knowledge from EHR data. Despite recent progress, current phenotyping approaches are manual, time‐consuming, error‐prone, and platform‐specific. This results in duplication of effort and highly variable results across systems and institutions, and is not scalable or portable. In this work, we investigate how the nascent Clinical Quality Language (CQL) can address these issues and enable high‐throughput, cross‐platform phenotyping.

**Methods:**

We selected a clinically validated heart failure (HF) phenotype definition and translated it into CQL, then developed a CQL execution engine to integrate with the Observational Health Data Sciences and Informatics (OHDSI) platform. We executed the phenotype definition at two large academic medical centers, Northwestern Medicine and Weill Cornell Medicine, and conducted results verification (n = 100) to determine precision and recall. We additionally executed the same phenotype definition against two different data platforms, OHDSI and Fast Healthcare Interoperability Resources (FHIR), using the same underlying dataset and compared the results.

**Results:**

CQL is expressive enough to represent the HF phenotype definition, including Boolean and aggregate operators, and temporal relationships between data elements. The language design also enabled the implementation of a custom execution engine with relative ease, and results verification at both sites revealed that precision and recall were both 100%. Cross‐platform execution resulted in identical patient cohorts generated by both data platforms.

**Conclusions:**

CQL supports the representation of arbitrarily complex phenotype definitions, and our execution engine implementation demonstrated cross‐platform execution against two widely used clinical data platforms. The language thus has the potential to help address current limitations with portability in EHR‐driven phenotyping and scale in learning health systems.

## 1 INTRODUCTION

Learning health systems (LHS) are organizations in which the delivery of care generates data and insights that can be analyzed and transformed into biomedical knowledge. This knowledge can then be used to improve the quality and efficacy of healthcare.^1^ A core aspect of generating this knowledge is the identification of patient cohorts in the electronic health record (EHR) matching certain clinical criteria, a process commonly referred to as EHR‐driven phenotyping. EHR‐driven phenotyping has applications across the continuum of LHS to conduct case‐control and cohort studies, clinical trial recruitment, clinical decision support (CDS), and quality measurement.^2^

We have established the Phenotype Execution and Modeling Architecture (PhEMA), an open‐source infrastructure to support clinicians, researchers, informaticists, and data analysts in standards‐based authoring, sharing, and execution of computable phenotype definitions.^3^ In this work, we continue to improve the PhEMA tools by proposing to adopt Clinical Quality Language (CQL),^4^ a Health Level Seven International (HL7) standard for formally representing logical expressions, as the computable phenotype representation. Our hypothesis is that if a standards‐based phenotype representation is used, it will enable execution across data platforms with a one‐time cost. That one‐time cost is the development of a CQL engine for each target platform, and this cost is preferable to manual phenotype translation, as it ultimately enables cross‐platform phenotyping at scale. We investigate whether this approach does enable cross‐platform phenotyping and demonstrate a newly built CQL evaluation engine that is able to execute CQL phenotype definitions against the Observational Health Data Sciences and Informatics (OHDSI) platform.^5^

We used a clinically validated phenotype definition for patients with heart failure (HF), a common, costly, and morbid condition affecting over 6 million United States adults and a high public health priority.^6^ The system was validated at multiple institutions and across data platforms, and is made available on GitHub to complement the current set of tools used by the observational research community, with the hope that our methods will contribute toward the future convergence of phenotyping systems.

## 2 BACKGROUND

In general, EHR‐driven phenotyping is a two‐step process: (a) defining the phenotype and (b) executing the phenotype. First, a phenotype definition must be developed, which is a resource‐intensive process involving multidisciplinary teams, and often requiring several iterations to produce a high‐quality, clinically valid result. Phenotype definitions typically consists of (a) clinical data elements of interest, such as demographics, medications, diagnoses, encounters, laboratory results, and other clinical observations, (b) lists of codes from published terminologies, called *value sets*, and (c) Boolean, aggregate, and temporal logical expressions that relate the data elements and value sets (phenotype *logic*). Additionally, the phenotype definition must be validated against a gold standard, most often derived from a resource‐intensive manual chart review.^7^, ^8^

Second, in order to assemble the cohort of interest, the phenotype definition must be *executed* against a clinical database. Without a directly executable standard representation, this involves human interpretation of a narrative description or flowchart illustrating the phenotype definition and translation into machine‐executable code, such as SQL or R. This is a time‐consuming and error‐prone process, which sometimes involves translating value sets into local terminologies.^9^, ^10^ Such phenotype definitions are not portable or scalable, as these steps must be repeated at every implementation site, resulting in duplication of effort and highly variable results.

In contrast, c*omputable* phenotype definitions are represented in an unambiguous formal language and can be executed against a database with minimal human intervention, reducing implementation effort and variability, increasing transparency, and enabling high‐throughput phenotyping.^11^ Two approaches enable computable phenotype definitions: common data models (CDMs) and dedicated phenotype logic execution environments. CDMs allow writing executable code that can be used against different clinical databases without code modifications. Research networks such as the OHDSI network, the Health Care Systems Research Network (HCSRN),^12^ Sentinel,^13^ the electronic Medical Records and Genomics (eMERGE) Network,^14^, ^15^, ^16^ the National Patient‐Centered Clinical Research Network (PCORnet),^17^ and the Accrual to Clinical Trials (ACT) Network,^18^ have used this approach with much success.^19^, ^20^ However, no single CDM is ubiquitous, and the code written for any given CDM is not executable against a different CDM. For example, the PCORnet CDM and the Observational Medical Outcomes Partnership (OMOP) CDM used by OHDSI both represent similar categories of medical data, however a query written against one cannot be directly executed against the other without modification because the schemas are different.

Logic representation standards like the healthcare‐focused Health Quality Measure Format (HQMF) and CDS Knowledge Artifact Specification (KAS), and general logic execution environments such as JBoss Drools and KNIME have also been shown to work in select use cases.^21^, ^22^ Software code is not based on any formal healthcare‐related standard, and while HQMF and CDS KAS show promise, they do not have human‐readable representations. General logic execution environments may present a significant implementation burden, with some institutions spending significant valuable resources and time, and still failing to get the systems running.^23^

Instead, clinicians, informaticists, and data analysts need an approach that allows them to collaborate with institutions using a variety of CDMs, and minimizes the number of times a phenotype has to be written. CQL is a formal logical expression language that supersedes HQMF and CDS KAS, and is intended to be used for electronic clinical quality measures (eCQMs) and CDS, as well as more general clinical knowledge representation use cases. The Centers for Medicare & Medicaid Services (CMS) and HEDIS (The Healthcare Effectiveness Data and Information Set) have published eCQMs using CQL. The emerging FHIR standard has also adopted CQL as one of its standard logical expression languages.^24^ Additionally, there are several tools for authoring knowledge content in CQL, such as the CMS Measure Authoring Tool (MAT)^25^ and the Agency for Healthcare Research and Quality's (AHRQ) CDS Connect authoring tool.^26^

CQL is organized into *libraries*—comparable to programming packages—which have the added benefit of being reusable across multiple CQMs and CDS. CQL has both a high level, human‐readable representation, and an equivalent machine‐readable representation, called the Expression Logical Model (ELM). The ELM is an abstract system tree (AST) representation of the language and has both a JSON and XML format. The intention of the language authors is that engine developers should focus on the evaluation of core logic expressed in the ELM, and use existing tools for parsing, expression simplification, and semantic analysis.^4^ Furthermore, CQL is data model agnostic, meaning that different data models, such as FHIR, OMOP or the Quality Data Model (QDM), may be utilized with the same logical constructs.

## 3 METHODS

### 3.1 Phenotype selection and translation

We adopted a pre‐existing HF phenotype definition that has been executed and clinically validated against multiple EHRs and sites.^27^, ^28^, ^29^ The HF phenotype definition uses several different data modalities, including demographic data, clinical diagnoses, clinical encounter types, as well as procedure orders. It also uses Boolean logic, temporal logic in the form of patient age and co‐occurrence of diagnosis with encounters, and an aggregate function. Additionally, it references a number of common clinical terminologies, including the International Classification of Diseases versions 9 (ICD‐9) and 10 (ICD‐10), the Current Procedural Terminology (CPT), as well as the Systematized Nomenclature of Medicine (SNOMED).

We began by representing the HF phenotype definition as a CQL library. We selected the FHIR data model for data element references because mappings already exist from the FHIR data model to many popular CDMs,^30^ and many CQL engine implementations support FHIR.^31^

**FIGURE 1:**
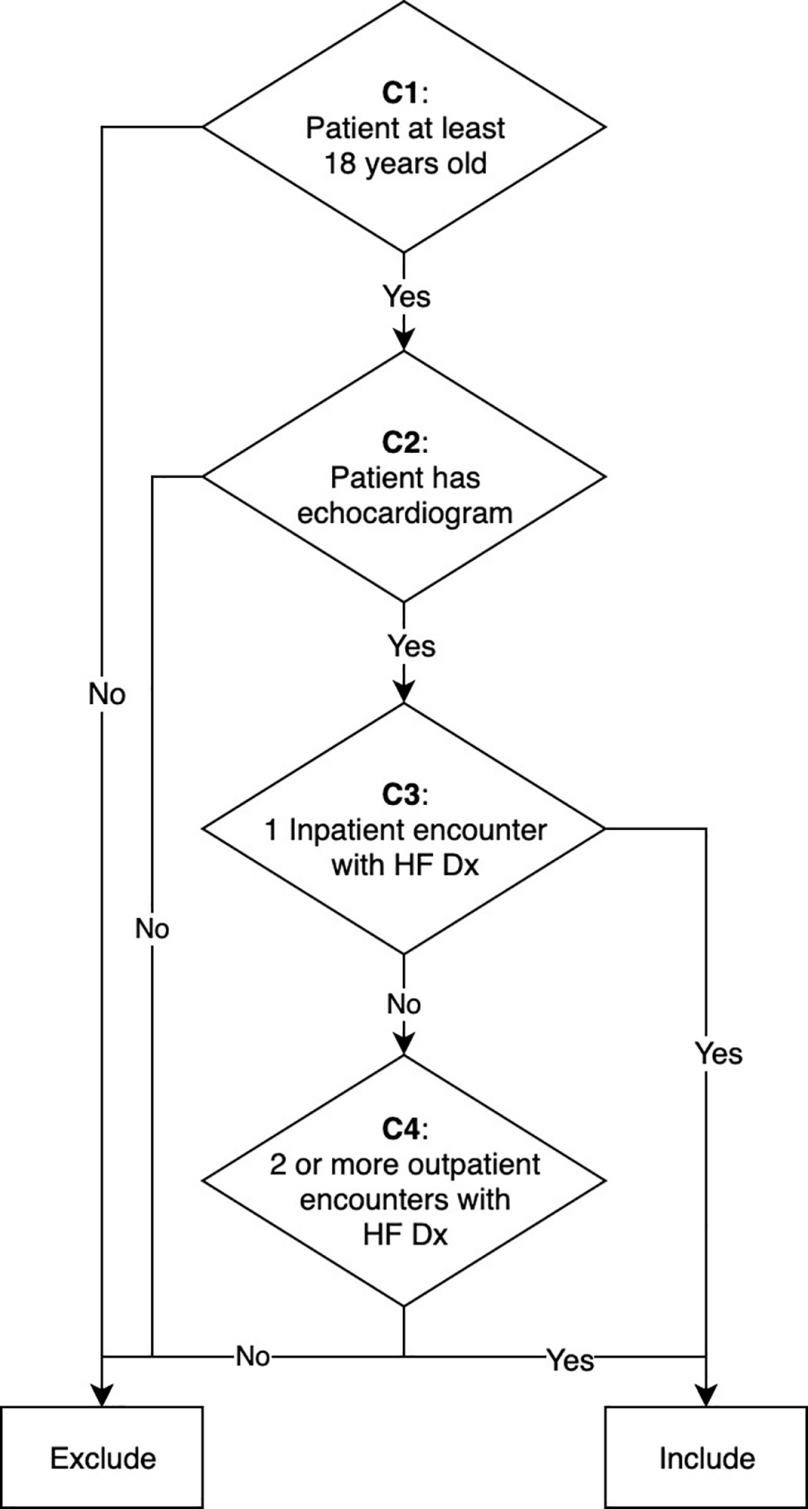
The HF phenotype definition. All criteria are labeled **C1** through **C4**. HF, heart failure

The HF phenotype logic is shown in Figure 1. Criteria **C1** and **C2** are mandatory, and the patient must also match either **C3** or **C4** to be considered a case. CQL is sufficiently expressive to represent these criteria, and the source, available in the project's GitHub repository^32^ has six total statements. One for each criterion, one to represent the disjunction of **C3** and **C4** (C*), and one to represent the final conjunction: C1 AND C2 AND C*.

Two value sets were needed, one for the HF diagnosis codes (Dx VS), which came from three different terminologies (ICD‐9, ICD‐10, and SNOMED), and one for the echocardiography procedure codes (Echo VS), from the CPT terminology. We used an existing Dx VS from the Value Set Authority Center (VSAC),^33^ which is also used by CMS for their HF eCQMs. We created and published a new Echo VS in VSAC. For inpatient and outpatient encounter types, we used individual codes from the ActCode^34^ terminology, as recommended by the FHIR standard.^35^

### 3.2 CQL engine development

**FIGURE 2:**
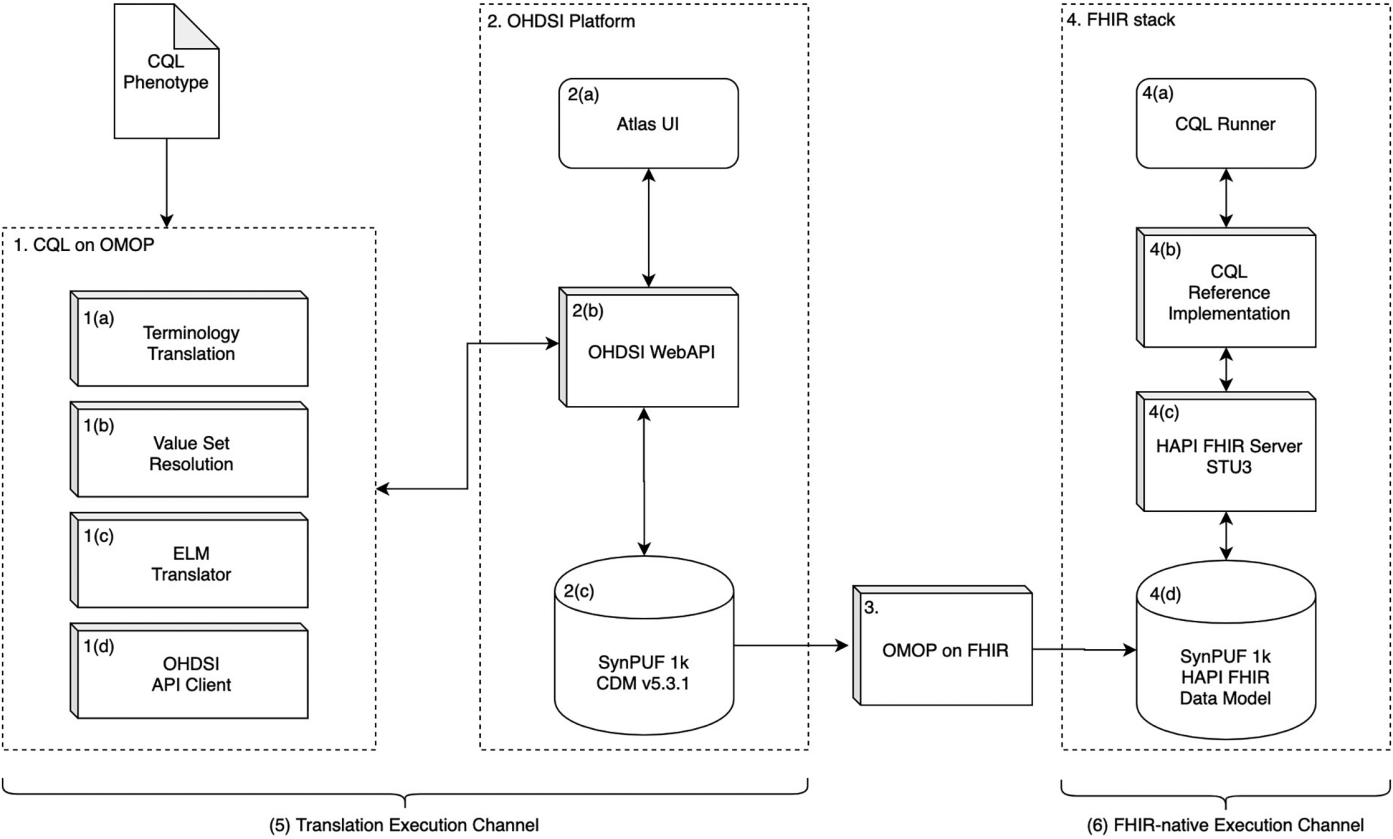
Experimental architecture. Box (1) shows the developed CQL on OMOP engine, box (2) the OHDSI data platform, box (3) the OMOP on FHIR data transformation tool, and box (4) the FHIR‐native stack used for cross‐platform validation. Box (1) shows our newly developed software, while boxes (2) to (4) are existing systems we leveraged. Pipelines (5) and (6) show the two validation methods. Both NM and WCM used the pipeline (5) architecture with their own data for phenotype execution. CQL, Clinical Quality Language; FHIR, Fast Healthcare Interoperability Resources; NM, Northwestern Medicine; OHDSI, Observational Health Data Sciences and Informatics; OMOP, Observational Medical Outcomes Partnership; WCM, Weill Cornell Medicine. [Correction added on 2 September 2020, after first online publication: Figure 2 has been revised.]

We chose to develop a CQL engine, called *CQL on OMOP* (Figure 2, box 1), for the OHDSI data platform. In addition to its use of the OMOP CDM, OHDSI has existing phenotype definition analysis and visualization tools built upon a Web application programming interface (API), making it possible to validate our results using independent methods. The OHDSI platform represents phenotype definitions in a transportable JSON format, and executes them using a library called *Circe* ^36^ that provides entities for representing phenotype logic, for example, **CriteriaGroup** and **DemographicCriteria**. CQL on OMOP translates a CQL‐based phenotype definition into the Circe representation and then uses the OHDSI Web API to generate the cohort (Figure 2, box 2[b]).

The engine was developed as an open‐source Java application^37^ and uses libraries provided by the CQL authors to parse CQL and generate an ELM tree.^38^ Entities from the Circe library are used to represent cohort criteria in the format expected by the OHDSI Web API. CQL on OMOP runs as a standalone application and can be configured to connect to an instance of the OHDSI Web API. Both CQL and ELM are supported as inputs, as well as value sets in several different formats, including the format produced by VSAC. Finally, the tool is developed in a modular way that makes it easy to add new CQL language features and support new data element correlations.

The process of value set resolution leverages CSV files downloaded from VSAC that were packaged with the HF CQL. The process of resolution (Figure 2, box 1[b]) matched value set object identifiers (OIDs) within these files and the CQL code. CQL on OMOP used the OHDSI Web API to identify the relevant concepts from each value set and build the OHDSI concept set (Figure 2, box 1[d]). The OHDSI platform primarily makes use of standard terminologies, with one exception being internal codes defined by OHDSI for visit types. This required us to implement a simple terminology translator between FHIR encounter types and OHDSI visit types (Figure 2, box 1[a]).

The core contribution of the CQL on OMOP engine is the ELM logic translator (Figure 2, box 1[c]). The engine implements a rule‐based, recursive descent language translation algorithm.^39^ In this algorithm, each node of the AST (ELM) is visited during a post‐order tree traversal and is translated based on a set of rules. To support the logic necessary to execute the HF phenotype definition, we implemented rules for Boolean conjunctions (**AND**) and disjunctions (**OR**), temporal logic to calculate patient age, numeric comparison, the **Count** aggregate function, filtering data by value sets, and correlated queries, which express relationships between data elements. Data model translation is performed during the creation of Circe criteria from ELM **Query** constructs.

### 3.3 Validation

The CQL on OMOP tool was validated in two ways—*cross‐institutional* and *cross‐platform*. First, the cross‐institutional validation checked the accuracy of the translated phenotype logic when executed on two instances of the same CDM (OMOP). This was done at Northwestern Medicine (NM) and Weill Cornell Medicine (WCM), and we verified the results manually to evaluate if the phenotype logic was correctly applied (Figure 2, pipeline 5). Second, we conducted a cross‐platform validation to evaluate that consistent results were returned when the same phenotype logic was applied to the same synthetic dataset in two different execution pipelines—an OMOP environment, and an independent, FHIR‐native CQL execution pipeline (Figure 2, pipeline 6).

#### 3.3.1 Cross‐institutional

The NM OMOP instance used for cross‐institutional validation is a subset of the patient population at NM, specifically, those consented for the eMERGE network, and is generated from the NM EpicCare EHR. The WCM OMOP instance includes the patient population at WCM and its affiliate NewYork‐Presbyterian (NYP) hospital with at least one recorded visit, condition, and procedure. In the outpatient setting, WCM physicians use the EpicCare EHR, and NYP uses the Allscripts Sunrise Clinical Manager for inpatient care.

Each institution selected a random set of 25 patients that were identified by CQL on OMOP as meeting the criteria of the HF phenotype (cases). Additionally, each institution selected 25 random patients who (a) were not included as a HF case, (b) had at least one echocardiogram procedure, and (c) had at least one relevant diagnosis code (noncases). In review of the HF phenotype definition, we believed the highest chance of error in the translation of the logic was in the portion aligning diagnoses with encounters (**C3** and **C4** in Figure 1). As this is a technical verification and not a clinical validation, we believed this would be more likely to identify implementation errors than a random selection of patients not meeting the case definition, as the majority of patients would simply be lacking diagnoses (given the overall expected low prevalence of HF). Cases and noncases were selected from the respective OMOP databases using SQL scripts that were prepared collaboratively by the reviewers ahead of time.

At NM, one reviewer (LVR) evaluated the set of 50 cases and noncases in OMOP. A random subset of 10 patient records (five cases and five noncases) was conducted by a second reviewer (JAP). At NM, each reviewer used the ReviewR tool, which provides a graphical interface and filtering capabilities against an OMOP database.^40^ At WCM, a similar process was followed with a primary reviewer (ETS) reviewing all 50 patient records and a second reviewer (PA) reviewing a random subset of 10 patient records. WCM reviewers accessed the OMOP database via SQL queries to retrieve the data elements needed for the results verification. Both institutions used the same SQL code to generate the random list of patients for review and followed the same written protocol. This code and documentation are available in the project's GitHub repository. We calculated Cohen's kappa to measure inter‐rater reliability, and overall system performance using precision and recall.

#### 3.3.2 Cross‐platform

To assess cross‐platform performance, we compared CQL on OMOP to the reference implementation of the CQL engine provided by the language authors,^41^ running against a HAPI FHIR server.^42^ We used data for 1000 patients from the Centers for Medicare & Medicaid Services' (CMS) Data Entrepreneurs' Synthetic Public Use File (SynPUF 1 k).^43^ Although synthetic, the dataset is intended to be representative of a typical claims dataset collected by CMS. The dataset was transformed into the OMOP CDM schema using the extract transform load (ETL) tool provided by the OHDSI community,^44^ and transformed into the FHIR format using the OMOP on FHIR tool.^45^

We ran the HF phenotype definition using CQL on OMOP against an OHDSI instance containing the SynPUF 1 k dataset and generated the resulting cohort of patients. We then ran the same HF CQL using the CQL reference implementation against a FHIR server containing the same SynPUF 1 k data and compared the resulting patient cohorts. Performance (agreement between the two systems) was measured using Cohen's kappa.

## 4 RESULTS

### 4.1 Cross‐institutional

**FIGURE 3:**
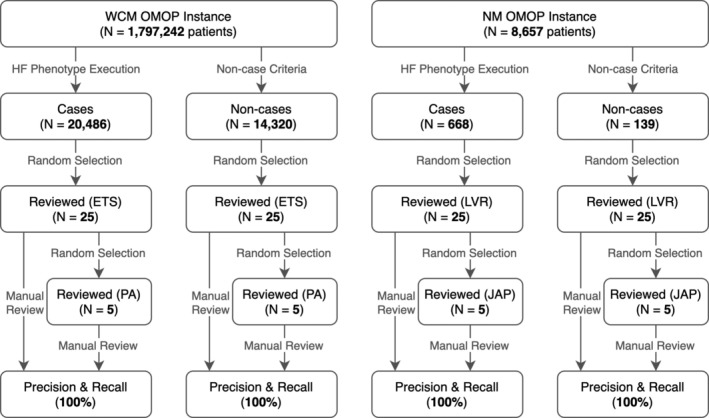
Results and validation flowchart for translation execution pipeline

The NM OMOP instance contained 8657 patients, of which 668 patients (7.7%) were identified by the HF phenotype definition, from which 25 were randomly selected for review. Of the 7989 patients not qualifying for the HF cohort, 139 patients had at least one HF diagnosis and at least one echocardiogram, from which 25 were randomly selected as the non‐case review cohort. Inter‐rater agreement was κ = 1.0 between the two reviewers, and the CQL to OMOP translation execution pipeline achieved both precision and recall of 100% (Figure 3).

Of the approximately 1 797 242 patients in the WCM OMOP instance, 20 486 (1.4%) were in the HF cohort. There were 14 320 patients that matched our non‐case criteria. The 25 cases and 25 noncases randomly selected for review demonstrated precision and recall of 100%. Inter‐rater agreement was again κ = 1.0.

### 4.2 Cross‐platform

After performing ETL on the SynPUF 1 k dataset, the resulting OHDSI instance contained 147 186 conditions, 55 261 visits, and 137 522 procedures for the 1000 synthetic patients. We confirmed the same counts of each data element after application of the OMOP on FHIR data transformation tool to verify no data were lost.

Running CQL on OMOP against an OMOP instance containing the SynPUF 1 k dataset resulted in a cohort with 94 members (9.4%). Executing the same CQL using the CQL reference implementation pointing to a HAPI FHIR server containing the same SynPUF 1 k dataset represented as FHIR resources also generated a cohort containing 94 patients. Since patient IDs were kept consistent by the ETL and OMOP on FHIR processes, we were able to confirm that these cohorts were in complete agreement with κ = 1.0.

## 5 DISCUSSION

We were able to express a HF phenotype algorithm as a CQL library, and demonstrate consistent execution across multiple institutions with different populations (NM and WCM), as well as different data platforms (OMOP and FHIR) representing the same synthetic patient population. Manual translation of the query logic was not required in this process, thereby, limiting the potential for error. Thus, CQL reduces duplication of effort, increases transparency and phenotype portability, and reduces variability. Furthermore, our selection of a clinically purposed language (CQL) will facilitate extension of this approach beyond research phenotypes to clinical and analytical needs of a LHS.

CQL libraries modularize logic using named statements and/or functions, facilitating reuse, which is highly beneficial for phenotyping as it enables defining cases and controls which often have shared logic. Well‐constructed libraries can then also extend past binary case‐control classification to include *suspected* cases, subphenotypes, related phenotypes, and even groups of phenotypes. Libraries can also be parameterized, which can be used to support local customization, within well‐defined bounds, to match site‐specific clinical and operational procedures.

Although our translation of the HF phenotype logic performed with high precision and recall, we note differences between the conceptual models used by CQL and the OHDSI Circe library. In Circe, phenotypes have specific entry and exit events, and the concept of *observation period* is used to determine cohort membership, which are not explicit concepts in CQL. Circe and CQL also have different internal AST representations. CQL uses a traditional AST with very simple nodes, and a topology correlated with the complexity of the represented logic, while Circe uses nodes that encode additional information, and generally has a simpler topology, only using the tree to encode conjunction and disjunction, occurrence count restrictions, and temporal correlations. CQL's more traditional AST structure lends itself well to language applications like translation and interpretation, while the structure of Circe may simplify SQL query construction and make it easier to build user interfaces to author cohort definitions. Lastly, criteria in Circe can be manually grouped into *inclusion rules*, which supports the generation of attrition statistics and visualizations. Since this grouping requires human intervention, it is not possible to generate meaningful inclusion rules in CQL on OMOP without introducing further conventions (eg, annotations), which we decided against to ensure cross‐platform support for CQL‐based phenotypes.

Using the FHIR data model for data element references resulted in several advantages. Due to the popularity of FHIR, a data model translation already existed for the OMOP CDM, which reduced the amount of implementation work necessary for CQL on OMOP. The HF phenotype logic references unambiguous data elements such as conditions and procedures, which are highly mature entities in the FHIR specification, and have very clear mappings to the OMOP CDM. However, some phenotype definitions may reference more nuanced data elements that may be more difficult to translate. While it may take more upfront work to deal with these issues in the CQL engine, this work will only have to be done once per target platform, reducing overall work required for phenotyping, along with phenotype variability.

The reduced expressiveness of Circe, compared to CQL, limited our current CQL on OMOP implementation. As many institutions within the OHDSI community develop cohort definitions using SQL, R, or other languages as opposed to Circe, this may have been a purposeful limitation by the OHDSI developers. CQL approaches the expressiveness of a general‐purpose programming language, and as such can express arbitrary arithmetic, and has many aggregate functions not supported by Circe (eg, **Sum** and **PopulationStdDev**). To address these limitations Circe can either be extended to be more expressive, or CQL on OMOP could bypass Circe and the OHDSI API entirely and access the database directly. While the latter approach would enable the full expressivity of CQL, the former approach is more desirable, since it maintains compatibility with all of the phenotyping and other tools in the OHDSI community.

An important limitation of the CQL language itself is that it is optimized for rule‐based logic using structured data elements, and does not explicitly define any mechanism for natural language processing (NLP) or integration with machine learning (ML) methods. Both of these techniques are important to the task of phenotyping,^46^, ^47^ and being limited to structured data and deterministic algorithms is a significant restriction. However, CQL does provide a mechanism to integrate with external systems using an approach called *foreign function invocation* (FFI). FFI enables a given engine implementation to make functions available to the CQL library author that execute code in an arbitrary environment, such as an NLP or ML pipeline, and make the execution results available in CQL. Furthermore, CQL can leverage existing NLP systems that already utilize the FHIR standard to provide standardized models and normalization rules for integrating unstructured data.^48^ These features could be used to develop extremely high fidelity phenotypes that make use of the latest NLP and ML algorithms.

We acknowledge additional limitations within our work. First, our evaluation was performed using a single phenotype (heart failure), and does not include support for all operators within CQL at this time. We selected the HF phenotype definition given its use of multiple data elements (diagnoses, encounters, procedures, demographics), temporal logic, and aggregate functions (**Count**), which represents commonly used building blocks across other phenotype definitions. Second, we recognize that the validation of 50 cases and 50 noncases may be seen as minimal, and that our selection of noncases is not representative of all patients not identified by the HF algorithm as cases. Given that our focus was on a technical verification and not a clinical validation, we believe that our review allowed us to focus on the most probable sources of error. Third, the upfront cost of developing a CQL engine for a new target platform may be prohibitive, and potential implementers of the proposed approach would need to balance this cost against potential benefits. If the implementer has no desire to share or reuse existing phenotype definitions, or if cross‐platform phenotyping is not a requirement, then using existing query tools may be more appropriate.

Despite the above limitations, we have shown that CQL can be used to represent and execute a clinically validated phenotype, using our CQL on OMOP engine. Due to its highly expressive nature, CQL could be used to represent longitudinal phenotypes with highly complex data relationships. Furthermore, in our experience, the CQL language specification (with its canonical AST) makes implementing language engines against arbitrary data platforms relatively easy. Therefore, CQL is a promising candidate as a formal phenotype representation standard that supports cross‐platform execution.

## 6 CONCLUSIONS

The task of EHR‐driven phenotyping is critical to biomedical knowledge generation, which supports the learning health system. Current techniques suffer from portability and scalability issues, requiring human intervention. This leads to errors, variability, lack of transparency, and greatly reduces potential throughput. To address these issues, we investigated CQL as a candidate language for representing clinical phenotype definitions, and demonstrated execution against multiple data platforms without local customization. We believe this approach could speed up phenotyping, regardless of the underlying data platform. Using a computable standard representation would also reduce duplication of work and potential for human error, and enable the large scale phenotyping needed for learning health systems.

In future iterations of the PhEMA project we plan to extend CQL language support in CQL on OMOP, translate additional clinical phenotypes into CQL, use CQL‐based phenotype definitions in clinical research studies, and extend existing phenotype authoring tools to generate CQL. Furthermore, we plan to develop CQL execution engines against other data platforms, such as the Informatics for Integrating Biology and the Bedside (i2b2) platform,^49^ and extend CQL to support NLP and ML. We will continue this work with existing phenotyping communities to publish methods and tools with the ultimate goal of convergence on a unified system to support high‐quality and high‐throughput phenotyping efforts.

## CONFLICT OF INTEREST

The authors declare no conflicts of interest.
